# The effects of position on gastric residual volume of premature infants in NICU

**DOI:** 10.1186/s13052-018-0591-9

**Published:** 2019-01-08

**Authors:** Alireza Khatony, Alireza Abdi, Batol Karimi, Abbas Aghaei, Hamidreza Saeidi Brojeni

**Affiliations:** 1 Social Development and Health Promotion Research Center, Kermanshah University of Medical Sciences, Kermanshah, Iran; 2 Students Research Committee, Kermanshah University of Medical Sciences, Kermanshah, Iran; 3 Nursing Department, School of Nursing and Midwifery, Doulat-Abad Street, Kermanshah, 6718996511 Iran; 4 Social Determinants of Health Research Center, Research Institute for Health Development, Kurdistan University of Medical Sciences, Sanandaj, Iran; 5 Clinical Research Development Center, Imam Reza Hospital, Kermanshah University of Medical Sciences, Kermanshah, Iran

**Keywords:** Residual volume, Premature, Infant, Enteral nutrition

## Abstract

**Background:**

Nutrition cares are of the main measures to save premature infants. In this regard, proper positioning is one of the key measures that is done by nurses; still there is a paucity of studies in this field and the results of these few studies are an area of ongoing debates. In light of this, the present paper is an attempt to determine the effects of different positioning on gastric residual volume in premature infants in NICU.

**Methods:**

A clinical trial cross-over study was carried out on premature infants in NICU. The subjects, who had inclusion criteria, were selected through convenience sampling based on inclusion criteria and randomly allocated into three groups. Gastric residual volume before and one hours after feeding was measured and recorded for three positions including right-lateral, left-lateral, and prone. The data was analyzed via SPSS-21 using descriptive statistics such as mean, standard deviation, and frequency; and inferential statistics such as Chi Squared, Kruskal Wallis test, and Friedman test.

**Results:**

Totally, 135 infants in three groups were studied and the results showed that minimum and maximum gastric residual volumes were in prone (6.49 ± 8.25 ML) and supine (12.59 ± 11.9 ML) positions, respectively. However, Kruskal Wallis test did not show a significant relationship between the three positions under study and the mean gastric residual volume.

**Conclusion:**

Prone position was featured with the lowest gastric residual volume and highest possibility of absorbing nutrient. Still, given the fact that no significant difference was found in the three groups, further and deeper studies are needed.

**Trial registration:**

The project is approved by Iranian Registry of Clinical Trial with no. IRCT. 201404134736 N6.

## Background

More than 15 million premature infants are born in the world every year. That is, one out of every 10 infants is born as a premature infant. This rate in Iran is 10–15% of live births. Fortunately, more than three-fourth of the premature infants can be saved provided that proper and accurate health cares are available [1]. The World Health Organization defines premature infants as the infants born before the 37th week from the first day of the last menstruate period and need more attention comparing with normal infants [2].

Due to anatomic and functional limitations and premature nervous function of their digestive system, premature infants suffer from digestive problems. Peristalsis moves of esophagus that leads food toward the stomach, are not properly developed in the premature infants, which indicates necessity of nutritional supports for these patients. Nutrition condition of these patients is measured based on gastric residual volume, which is the amount of residual food from the last meal when the next meal begins. It is a measurable parameter that indicates volume of gastric emptying and nutrition capacity. The parameter is measured before a meal and includes gastric acid and enzymes [1].

Proper enteral nutrition in premature infants decreases mortality rate and spread of infection, improves weight gain, and also shortens hospitalization term [3]. On the other hand, poor nutrition bearing prolongs hospitalization [4]. Gastric emptying depends on a variety of factors such as type of milk (milk power or mother’s milk), volume of milk, and physical condition [5]. Positioning the infant in the proper position is one of the nurses’ main tasks [3], and doing it perfectly needs more reliable evidences and clues [6].

Different positions have different effects on the premature infants; accordingly, prone position increases arterial O2 saturation, improves respiration and rib cage motions, decreases apnea in the infants with a history of apnea, improves sleeping, and attenuates regurgitation. However, this position increases orthopedical disorders and delays muscles development. On the other hand, infants are prone to sudden infants’ death syndrome. Therefore, the infants positioned in prone position should be under cardiopulmonary and arterial o2 saturation monitoring. On the bright side, prone position notably decreases the number and severity of regurgitation and gastric residual volume one hours after a meal [7].

Some studies have shown that gastric residual volume one hour after a meal in right-lateral position is less than that in left-lateral and prone positions [8]. However, Hewida Ahmed reported that right-lateral and supine positions were not effective in gastric residual volume one hour after the meal in 35 infants of NICU [9]. Moreover, Cohen et al. in the USA reported that gastric residual volume three hours after gavage in prone, supine, right-lateral, and left-lateral positions was not significantly different [5]. Chen et al. maintained that gastric residual volume in prone position was less than that in right-lateral position [10]. A review study by Smith (2011) showed that gastric residual volume one hour after the meal in right-lateral position was significantly less than that in left-lateral position; the residual volume in prone position was less than that in left-lateral position; and the residual volume in prone position was less than that in right-lateral position. Still, they found no significant difference three hours after the meal [8].

Despite the fifty years history of research works on nutrition in premature infants, there is no broad agreement about the best body position after a meal, and the results of the studies are an ongoing area of debate [11]. Given the need for more reliable evidences about the best position(s) after meal in premature infants, the present study is aimed at determining the effects of body position on gastric residual volume in premature infants in NICU.

## Methods

The study was carried out as a clinical trial in 2016. The population was the premature infants hospitalized in NICU who were fed through gavage, the premature baby is defined based on the gestation age < 37 weeks [12], and in this study the subjects were 28–36 gestational age old. The subjects were hospitalized in Kermanshah Imam Reza Hospital with 18 active beds. Based on mean and standard deviation of gastric residual volume reported in Ahmad Hoveida [9], test power of 90%, type I error of 0.05 and the sample size formula for interventional studies, number of participants was determined equal to 123, and taking into account 10% dropout, 135 participants were selected.

Inclusion criteria included consent of the parents, pregnancy term ranged from 28 to 36 weeks, mean Apgar score at birth higher than 6, stable physiological indices (heart rate, blood pressure, respiratory rate, O2 saturation) checked by a neonatologist, gavage feeding, and feeding mother’s milk. As to exclusion criteria, lack of parents’ consent, development of intraventricular hemorrhage, having necrotizing enterocolitis, having congenital malformations and digestive problems as confirmed by a specialist, pneumothorax, convulsion, intolerance of feeding, unstable vital signs, and need for mechanical ventilation are notable. Moreover, breast milk intolerance and the physician’s order to stop feeding were other exclusion criteria.

Data gathering tool was a form with two sections; section one was about demographics of the participants (e.g. weight, gender, pregnancy age, apgar score, type of delivery, cause of hospitalization, and positioning); and section two was to record gavage-fed milk volume. To examine content validity of the tool, it was provided to 12 faculty board members in Kermanshah University of Medical Science and modified based on the feedbacks. To record personal information and medical record of the infants, medical file and birth ID card were used. Standard syringe (5 cc SUPA Co.) was used to measure gastric residual volume.

**Fig 1:**
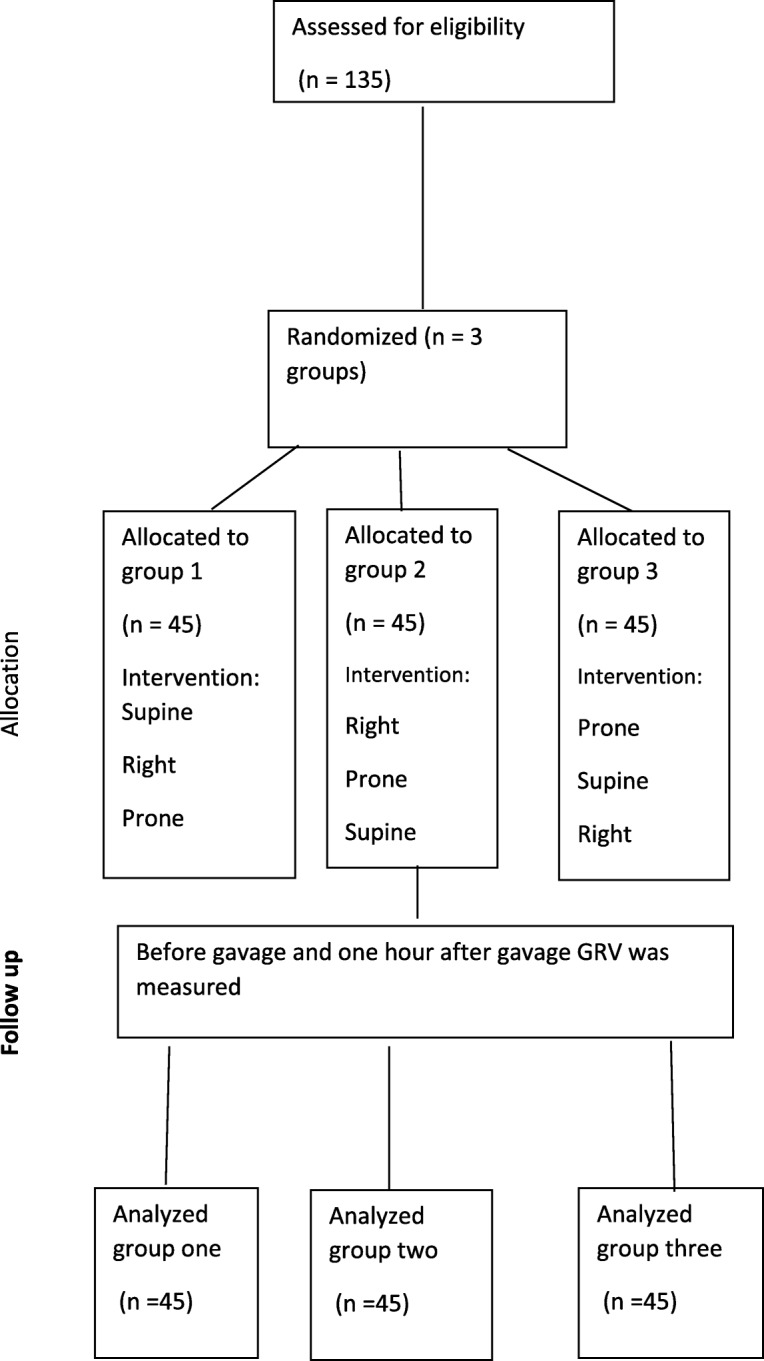
flow chart of Consort for recruiting, intervention and follow up of the study As the above figure shows, the 135 patients randomized to three groups, each group positioned in three states, and GRV was measured before and one hour after gavage, and then the data were analyzed

After securing the required permissions from the Department of Research and Technology, Kermanshah University of Medical Science and the officials of Imam Reza Hospital, the authors attended the NICU and selected the subjects who met the criteria. To this end, the parents were briefed at first about the objectives and procedure of the study and asked to sign an informed letter of consent. The infants were selected using convenience sampling and then randomly grouped into three groups using random number tables. The subjects in each group were positioned in supine, right-lateral, and prone positions for one hours so that the subjects in group one were positioned in supine, right-lateral, and prone positions; subjects in group two were positioned in right-lateral, supine, and prone positions; and subjects in group three were positioned in prone, supine, and right-lateral positions. Before gavage feeding, gastric residual volume was measured, based on the ward routine and related evidence [13], and then it was reinterred into the stomach, after that the subjects received their mother’s milk. The milk was given through bottle into gavage syringe. Gastric residual volume was also measured one hour later (Fig. 1).

The collected data was analyzed in SPSS-21 using descriptive (simple and relative frequency, mean, and standard deviation) and analytical statistics including intergroup and intragroup analyses via chi square, Kruskal Wallis, and Friedman tests. To examine normality of the quantitative variables, Shapiro Wilk test was used. (*P*-value = 0.05).

A letter of permission with ethics code Kums.rec.1394.279 was secured from Ethics Committee of the University and the proposal was registered in Iran Clinical Trial database under the No.:IRCT.201404134736 N6. Moreover, a letter of introduction was secured from the Faculty of Nursing and Midwifery of the university and submitted to the head of Kermanshah Imam Reza Hospital. As noted, an informed letter of consent was signed by the parents and they were ensured about confidentiality of their data and anonymity.

## Results

In this study, 135 infants in three groups were studied. There were 79 (58.5%) boys in the sample group and mean and standard deviation of age and weight of the infants and pregnancy age of the mothers were 5.63 ± 3.99, 1750 ± 533, and 32.08 ± 2.34 respectively. Majority of the subjects were in NICU due to respiratory failure (*n* = 56; 41.5%) and prematurity (*n* = 54; 40%), 89.6% of infants (121 individuals) had been born through caesarian section and apgar score of 59 infants (43%) was estimated equal to eight. The subjects were examined in terms of the variables pregnancy age of the mother and apgar score and no significant difference was observed. Still, there was significant difference in terms of age, gender, weight, reason for hospitalization, and delivery method (Table 1).

**Table 1:** Demographics based on the study groups

Groups Quantitative variables		Group one Mean (standard deviation)	Group two Mean (standard deviation)	Group three Mean (standard deviation)	Total Mean (standard deviation)	Statistical test
Age (day)		6.13 (4.15)	4.69 (3.90)	6.09 (3.82)	5.63 (3.99)	×2 = 7.36* P = 0.025**
Pregnancy age (week)		31.67 (2.26)	32.64 (2.24)	31.8 (2.45)	32.08 (2.34)	×2 = 4.65* P = 0.097
Infant’s weight (gr.)		1664.44 (537.10)	1954.22 (573.23)	1633.33 (432.36)	1750.67 (533.89)	×2 = 8.96* P = 0.011**
Groups Qualitative variables		Group one Mean (standard deviation)	Group two Mean (standard deviation)	Group three Mean (standard deviation)	Total Mean (standard deviation)	Statistical test
Apgar score	7	13 (28.9)	24.4))11	17.8))8	23.7))32	=6.164 *P* = 0.405
	8	20 (44.4)	33.3))15	53.3))24	43.7))59	
	9	(22.29)10	28.9))13	22.2))10	24.4))33	
	10	4.4))2	13.3))6	6.7))3	8.1))11	
	Total	(100)45	(100)45	100))45	100))135	
Gender	Boy	35.6))16	75.6))34	64.4))29	58.5))79	P < 0.001**
	Girl	64.4))29	24.4))11	16.6))16	41.5))56	
Cause of hospitalization	Prematurity	24.4))11	57.8))26	37.8))17	40))54	=14.4 P = 0.025**
	Respiratory failure	60))27	22.2))10	42.2))19	41.5))56	
	Multiple pregnancy	6.7))3	8.9))4	8.9))4	8.1))11	
	Sepsis	4 (9.8)	11.1))5	11.1))5	10.4))14	
Delivery	Natural delivery	20))9	2.2))1	8.9))4	10.4))14	7.81= P = 0.02**
	Caesarian section	80))36	97.8))44	91.1))41	89.6))121	

*is related Kruskal-Wallis H Test

**is significant

Based on Shapiro Wilk test, the variables infants’ age (*p* < 0.001), pregnancy age (p < 0.001), and infant’s weight (*p* = 0.006) were not normally distributed. In terms of mean gastric residual volume, *p*-value was 0.001, which indicates that the variable is not normal. This means that nonparametric tests should be used to examine the variables.

There was no significant different between the three positions in term of gavage volume. The first lavage was also not different in group1 and 2; however in group 3, lavage volume was higher before positioning in prone position (K2 = 15.37, *P* < 0.001) (Table 2).

**Table 2:** Comparing first lavage and gavage volume of groups in three position using Friedman test

Groups		First lavage Mean (SD) ML	Gavage volume Mean (SD) ML
Group 1	supine	0 (0)	9.85 (6.03)
	Right lateral	0.04 (0.29)	9.92 (5.99)
	prone	0.44 (0.29)	9.98 (6.04)
Statistical test		K2 = 1.00, *P* = 0607	K2 = 2.00, *P* = 0.368
Group2	right	0 (0)	6.28 (3.65)
	supine	0.1 (0.07)	6.40 (3.57)
	prone	0.12 (0.59)	6.62 (3.45)
Statistical test		K2 = 2.00, P = 0.368	K2 = 4.00, *P* = 0.135
Group3	prone	0.61 (0.84)	7.86 (5.12)
	supine	0.14 (0.34)	7.91 (5.09)
	right	0.14 (0.43)	7.095 (5.08)
Statistical test		K2 = 15.37, P < 0.001*	K2 = 4.00, P = 0.135

Mean gastric residual volume before gavage and in supine position in group one and three was higher than that in right lateral and prone positions; however, Kruskal Wallis test showed that mean gastric residual volume was not significantly different based on the position. Intergroup analyses showed that only in the group three, gastric residual volume in right-lateral and prone positions was significant less than that in prune position. (Table 3).

**Table 3:** Mean and standard deviation of gastric residual volume in different positions based on intergroup and intragroup point of view

Groups Position	Group one Mean (standard deviation) ML	Group two Mean (standard deviation) ML	Group three Mean (standard deviation) ML	Statistical test
Supine	1.21 (2.09)	0.46 (1.06)	0.92 (1.18)	**P = 0.047 ×2 = 6.10
Rightlateral	0.91 (1.68)	0.56 (1.06)	0.34 (0.86)	**P = 0.232 ×2 = 2.92
Prone	0.58 (1.27)	0.53 (1.47)	0.40 (1)	**P = 0.610 ×2 = 0.989
Statistical test	***×2 = 1.10 *P* = 0.577	***×2 = 2.28 *P* = 0.318	***×2 = 10.34 *P = 0.006	

*is significant

**Kruskal Wallis test

***Friedman test

## Discussion

Despite the fact that gastric residual volume in prone and right-lateral positions was less than that in supine position (except for group two), Kruskal Wallis test showed no significant difference among the three groups in this regard. Intragroup survey using Friedman test showed that gastric residual volume in the third group and in prone and right-lateral position was significantly less than that in prone position. Different studies have reported inconsistent results; Sungerz et al. (2013) conducted a prospective study titled “surveying the effects of body position on gastric residual volume” and found a significant difference in terms of gastric residual volume and body position. They reported lower gastric residual volume in right-lateral and prone positions comparing with supine and left-lateral positions [14]. Their study is consistent with present one with regard to the fact that the lowest gastric residual volume was observed in prone and then right-lateral positions; while the studies are inconsistent as we found no significant relationship between these positions. Although, there was no statistically significant relationship between position of the infants and residual gastric volume, the difference was clinically significant and the lowest and highest residual volumes were observed in prone and supine positions respectively. Thereby, prone position after milk gavage can be effective in attenuating gastric residual volume in premature infants. Chen et al. (2013) examined the effects of body position on gastric residual volume in premature infants. They positioned the subjects in supine position for three hours and then in prone position for another three hours and vice versa and then measured gastric residual volume 30, 60, 90, and 120 min after gavage. They reported that gastric residual volume after gavage in prone position was significantly lower than supine positions at all measurement points (*P* < 0.001); while emptying rate at first 30 and 60mins after feeding in prone position was higher than that of supine position [10]. Jebraieli et al. (2008) studied 100 premature infants and reported that gastric residual volume in prone and right-lateral positions decreased significantly one and two hours after gavage (*p* < 0.001). In addition, they noted that these two positions had similar effects on gastric residual volume and mean gastric residual volume in these two positions was not significant. Thereby, they argued that all the infants remained in these two positions suffered no side-effect. Given that these two positions had similar effects, both of them can be used after feeding the infants [15]. Their results in terms that right-lateral and prune positions decreased gastric residual volume, while the difference was not significant, are consisted with the present study.

Different results have been reported by other studies. Hussein Howeida (2012) studied the effects of right-lateral and semi-fowler’s positions after feeding on gastric residual volume in infants in NICU and reported that there was no significant relationships between these two positions. Still, since the both positions were effective on decreasing the residual volume, they are recommended after feeding infants [9]. Hawang et al. (2003) (3) measured gastric residual volume in different positions (prone, left-lateral, supine, right-lateral, full right-lateral, and full right-lateral recumbent). They reported that gastric residual volume was the lowest in right-lateral position (*p* < 0.05). Gastric residual volume also showed significant changes over time (*P* < 0.001) so that the authors believed that the best position to decrease gastric residual volume in premature infants after gavage was right-lateral. Although, the best positions to decrease gastric residual volume, according to some enteral nutrition guides, are supine and prone positions [16], results of a systematic review and review study by Smith (2011) on gastric residual volume in infants indicated that the residual volume was lower one hour after a meal in right-lateral and prone positions [8]. Inconsistent results can be explained by different sampling methods and demographical differences. For instance, there were significant differences among the three groups in some of demographical variables like weight, gender, cause of hospitalization and delivery method, and the literature showed that some variables such as gestational age, respiratory distress could be the predictors of gastric retention [17], and however there was no additional evidence about other variables. Moreover, it was not possible to use parametric tests to remove the effects of these intervening variables, since the variable gastric residual volume was not normally distributed. The results are influenced by these factors and this can be considered as a limitation of the study, so, it is suggested to conduct other studies.

## Conclusions

The prone position was featured with the lowest gastric residual volume and highest possibility of absorbing nutrient for premature infants. Still, given the fact that no significant difference was found in the three groups, further and deeper studies are needed.

## Abbreviations

KUMS: Kermanshah University of Medical Sciences
NICU: Neonatal Intensive care unit
